# Age- and Sex-Specific Risks of Major Cardiovascular Complications and All-Cause Mortality Following Elective Hip and Knee Arthroplasty in the Netherlands: A Dutch Hospital Data Registry Study

**DOI:** 10.1016/j.artd.2024.101597

**Published:** 2025-01-03

**Authors:** Mark J.R. Smeets, Maaike G.J. Gademan, Rob G.H.H. Nelissen, Suzanne C. Cannegieter, Banne Nemeth

**Affiliations:** a Department of Clinical Epidemiology, Leiden University Medical Center, Leiden, Zuid-Holland, The Netherlands; b Department of Orthopaedics, Leiden University Medical Center, Leiden, Zuid-Holland, The Netherlands; c Section Thrombosis and Hemostasis, Department of Internal Medicine, Leiden University Medical Center, Leiden, Zuid-Holland, The Netherlands

**Keywords:** Arthroplasty, Replacement, Hip, Arthroplasty, Replacement, Knee, Venos thromboembolism, Hemorrhage, Arterial thromboembolism, Mortality

## Abstract

**Background:**

Age and sex are well-known risk factors for cardiovascular complications and mortality following total hip arthroplasty (THA) and total knee arthroplasty (TKA). Yet, stratified up-to-date absolute risk estimates, which are needed to optimize prevention, are lacking.

**Methods:**

All Dutch patients who had a first primary THA and TKA for osteoarthritis between 2015 and 2021 were included. Data on baseline characteristics, procedures, and outcomes were obtained from the Dutch Hospital Data and Population registries, after linkage. Overall risks for venous thromboembolism, arterial thromboembolism, bleeding, and all-cause mortality were estimated at 30 and 90 days following surgery. Time trends were assessed by plotting 90-day risks by year. Lastly, 90-day risks were stratified by age and sex categories.

**Results:**

A total of 123,809 THA and 132,726 TKA patients were included. Females accounted for 63% and 65% of THA and TKA patients, respectively. At 90 days, overall risks were all below 1%. We observed no clear time trends in the risks over recent years. The stratified analysis showed that especially men older than 80 have a complication risk of at least 3%. Interestingly, the risk of venous thromboembolism and bleeding, following a THA, was observed to be relatively high in men and women in the youngest age category.

**Conclusions:**

Generally, the 90-day incidence of cardiovascular complications and all-cause mortality is low but can be at least 3% for men in the highest age category. With this knowledge, perioperative preventive measures can be targeted more precisely, and shared decision-making improved.

## Introduction

Total hip arthroplasty (THA) and total knee arthroplasty (TKA) are associated with serious postoperative cardiovascular complications, including venous thromboembolism (VTE), arterial thromboembolism (ATE), major bleeding and death [[1], [2], [3], [4]].

With the implementation of fast-track treatment protocols and enhanced recovery after arthroplasty surgery, over the last decades, overall estimates of cardiovascular complications and death have declined significantly [5,6]. However, as numbers of THA and TKA procedures are expected to rise with the aging population (in the United States, eg, 635,000 THA and 935,000 TKA procedures are projected for 2030), absolute numbers of cardiovascular complications will rise likewise and, therefore, have a major impact on society [7]. Hence, although relative risks seem to be low, absolute numbers are high, so continuous efforts have to be made to optimize prevention of these complications.

To further enhance prevention, risk estimates need to be specified on an individual patient level rather than the overall THA/TKA population. This is needed as the characteristics of patients, who undergo these procedures, are (increasingly) heterogeneous, and therefore, their risks of complications as well [[8], [9], [10]]. Examples of this increasing heterogeneity in the patient population are the wider age distribution (due to an increase of especially younger patients undergoing a THA or TKA) and the increase of patients who have more comorbidity [11]. In addition, it is well known that, independent of arthroplasty surgery, the risk of cardiovascular disease differs between men and women [12]. Yet, up-to-date age- and sex-stratified absolute risk estimates following THA/TKA are scarce. Most recent studies that investigated the association between age or sex and cardiovascular complications or mortality following a THA/TKA used logistic regression to determine odds ratios [[13], [14], [15], [16], [17]]. While these studies show that age and sex are associated with cardiovascular complications and mortality, an individual patient is not helped by the information that their odds of a complication are increased by X% because the baseline risk is unknown. To really individualize prevention, age- and sex-stratified absolute risk estimates are needed. Furthermore, these absolute risk estimates can be helpful to adequately counsel patients on their estimated complication risk.

Therefore, we aimed to describe overall risks, time-trends, and age- and sex-specific risks of cardiovascular complications and all-cause mortality over recent years (2015-2021) after a first primary THA or TKA procedure in the entire Dutch population.

## Material and methods

### Design and setting

In this nationwide cohort study, we used anonymized, non-publicly available, data from the Dutch Hospital Data (DHD) [18,19], the Population [20], the Population Death [21], and the Cause of Death [22] registries, which are managed by Statistics Netherlands. Data could be linked between registries based on a unique, anonymous, person identification number generated by Statistics Netherlands.

The DHD registry contains data on diagnoses and procedures performed in all hospitals in the Netherlands but lacks data on procedures performed in private clinics (between 2015 and 2021, the percentage of procedures performed in private clinics increased from approximately 5% to 20% for THA and 10% to 22.5% for TKA) [23]. Furthermore, the DHD registry is limited to diagnoses and procedures registered during a hospital admission (including those where a patient undergoes a procedure and is discharged on the same day) and emergency room visits exceeding 4 hours.

The Population registry contains information on sex and date of birth. The Population Death registry contains the date of death while the Cause of Death registry contains the registered cause of death.

### Population

Patients who underwent a primary THA or TKA procedure for the first time between 2015 and September 2021 were included to allow for 90-day follow-up, as data from 2022 onwards were not yet available. The year 2015 was chosen as the starting point because linkage between registries was complete from 2015 onwards.

Only primary procedures with the indication osteoarthritis were included. For each patient, only a first THA and TKA (ie, right or left) was included to avoid dependency between observations (ie, the similarity in complication risk is expected to be higher for 2 procedures in the same patient compared to 2 procedures for separate patients) [24]. To identify a previous contralateral procedure, we searched in the years that preceded the inclusion year, back until 2013. Because of a change in the quality of the data, the lookback for a previous procedure was not extended beyond 2013 [25]. Other exclusion criteria were age below 18 years at the time of surgery and receiving multiple protheses on the same day.

### Variables

The exposures of interest were THA and TKA for osteoarthritis (identification codes are provided in Appendix Table 1).

Outcomes of interest were VTE, ATE, bleeding, and all-cause mortality. VTE included pulmonary embolus (PE) and deep vein thrombosis (DVT) diagnoses. ATE included myocardial infarction, ischemic stroke, and transient ischemic attack diagnoses. Bleeding included gastro-intestinal bleeding, hemarthrosis, respiratory tract bleeding, intracranial bleeding, urinary tract bleeding, and procedural site bleedings. Each of these outcomes were obtained from the DHD and Cause of Death registry based on International Classification of Diseases, 10th Revision coding (Appendix Table 1).

### Missing data

Some of the medical records, registered by Statistics Netherlands, are incomplete in the sense that they lack a diagnoses or procedure code. For these records, Statistics Netherlands performs a single imputation based on a complete record, which most closely resembles the incomplete record [19]. Out of the included THA and TKA procedures, 0.19% of THA and 0.30% of TKA procedures were imputed by Statistics Netherlands. Similarly, out of the identified complications, 0.34% were imputed.

### Data analyses

Baseline pieces of information on age, sex, and year of surgery are reported as means with standard deviation or counts and percentages, as appropriate.

For our first aim, we estimated cumulative incidences (risks), with death as competing risk, and with 95% confidence intervals (CIs) for each of the outcomes over the inclusion years combined. The date of the THA or TKA procedure was the start of follow-up. The end of follow-up was either 30 or 90 days following the THA or the TKA separately.

For the second aim, to assess temporal trends in the risks for each of the outcomes of interest, 90-day cumulative incidences, with death as competing risk and with 95% CI, were estimated in each of the inclusion years and plotted for visual inspection.

For the third aim, patients were stratified on age and sex. Age was categorized in 5 categories: 18-49, 50-59, 60-69, 70-79, and 80+ years. In all categories, the 90-day cumulative incidence (including 95% CI) for each of the outcomes of interest was estimated, with death as competing risk, and plotted. Here, the overall risk ratios for sex at 90 days were also estimated for each of the outcomes.

All statistical analyses were performed using R version 4.1.3 (R Foundation for Statistical Computing, Vienna, Austria).

The study protocol was approved by the Scientific Committee of the department of Clinical Epidemiology, within our hospital. Because of the use of pre-existing, de-identified data, institutional review board approval was not required.

## Results

**Figure 1:**
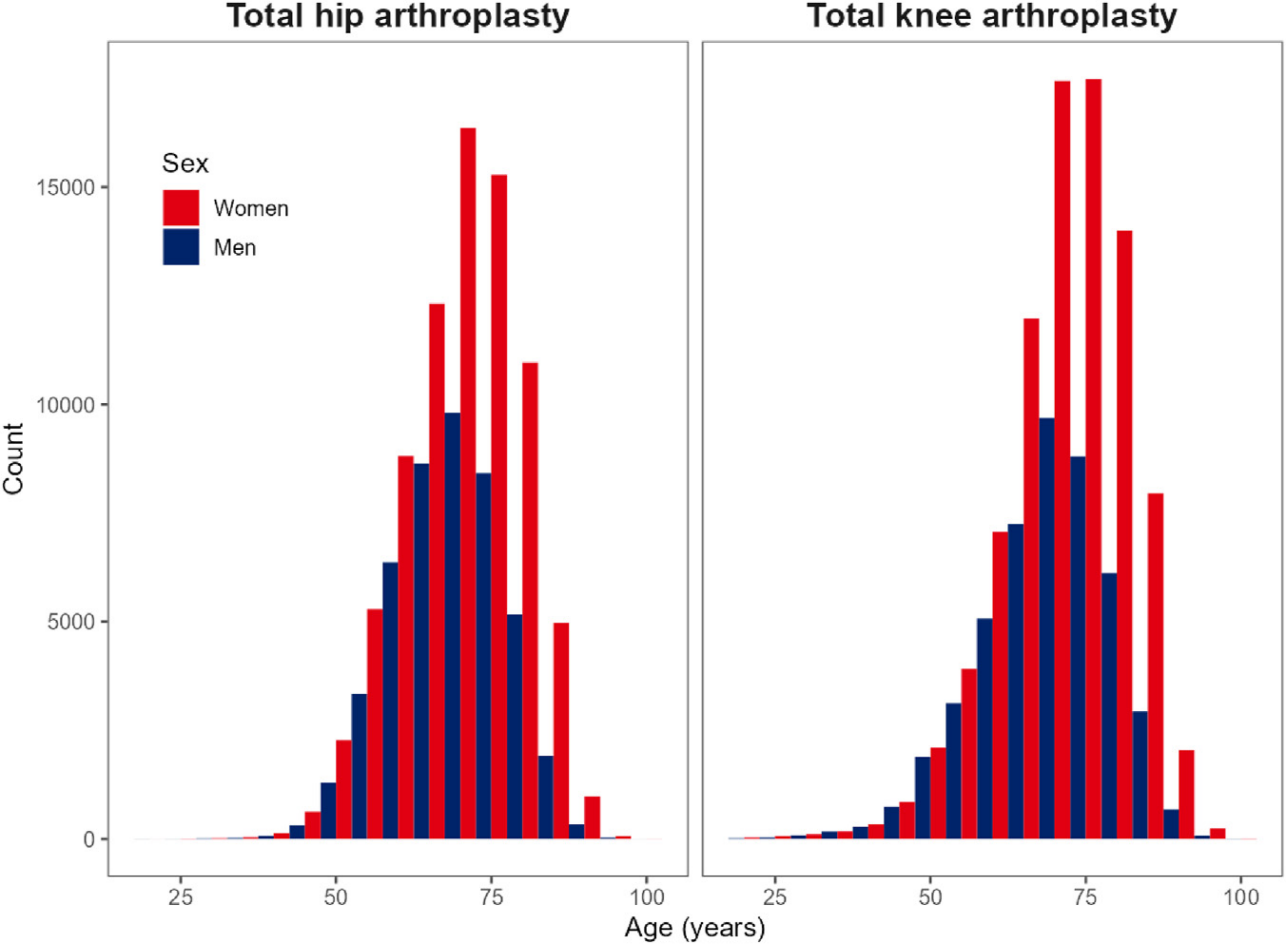
Age and sex distribution of included arthroplasty patients.

In total, 123,809 patients who underwent a THA were included in the study, as well as 132,726 who underwent a TKA. The mean age was 69 years and 71 years for THA and TKA patients, respectively. Furthermore, among THA patients, 63% were women, while for TKA, this was 65% (Table 1). Especially after the age of 70 years, there are approximately twice as many women undergoing a THA or TKA procedure compared to men (Fig. 1).

**Table 1:** Baseline characteristics.

Characteristics	Total (*N* = 256,536)	THA (*N* = 123,809)	TKA (*N* = 132,726)
Age, years (SD)	70 (9.7)	69 (9.1)	71 (10.1)
Female, *n* (%)	163,902 (64)	78,112 (63)	85,790 (65)
Procedures by year, *n* (%)			
2015	39,831 (16)	19,094 (15)	20,736 (16)
2016	39,979 (16)	19,036 (15)	20,943 (16)
2017	40,197 (16)	19,657 (16)	20,540 (15)
2018	40,594 (16)	19,845 (16)	20,749 (16)
2019	40,960 (16)	20,099 (16)	20,861 (16)
2020	30,111 (12)	14,552 (12)	15,559 (12)
2021	24,864 (10)	11,526 (9)	13,338 (10)

SD, standard deviation; THA, total hip arthroplasty; TKA, total knee arthroplasty.

### Overall risk estimates

For THA, the risk of VTE was 0.28% (95% CI 0.25-0.31) and 0.37% (0.34-0.40) at 30 and 90 days, respectively (Table 2). For ATE, these risks were 0.21% (0.18-0.23) and 0.35% (0.32-0.38). The 30-day bleeding risk was 0.77% (0.72-0.82), and the 90-day risk was 0.86% (0.81-0.91). Lastly, the risks of all-cause mortality following THA were 0.08% (0.07-0.10) and 0.19% (0.17-0.22).

**Table 2:** Cumulative incidence of cardiovascular complications and all-cause mortality at 30 and 90 days following THA or TKA over 2015-2020.

Complications	Number of events at 30 days (*n*)	Cumulative incidence % (95% CI) at 30 days	Number of events at 90 days (*n*)	Cumulative incidence % (95% CI) at 90 days
Total hip arthroplasty (N = 123,809)				
Venous thromboembolism	346	0.28 (0.25-0.31)	458	0.37 (0.34 - 0.40)
Pulmonary embolism	294	0.24 (0.21-0.26)	384	0.31 (0.28 - 0.34)
Deep vein thrombosis	63	0.05 (0.04-0.06)	93	0.08 (0.06 - 0.09)
Arterial thromboembolism	255	0.21 (0.18-0.23)	432	0.35 (0.32 - 0.38)
Myocardial infarction	109	0.09 (0.07-0.10)	184	0.15 (0.13 - 0.17)
Ischemic stroke	94	0.08 (0.06-0.09)	172	0.14 (0.12 - 0.16)
Transient ischemic attack	53	0.04 (0.03-0.05)	79	0.06 (0.05 - 0.08)
Bleeding	957	0.77 (0.72-0.82)	1066	0.86 (0.81 - 0.91)
Procedural site	829	0.67 (0.62-0.72)	866	0.70 (0.65 - 0.75)
Gastrointestinal	22	0.02 (0.01-0.03)	30	0.02 (0.02 - 0.03)
Respiratory tract	a	a	21	0.02 (0.01 - 0.02)
Intracranial	a	a	30	0.02 (0.02 - 0.03)
Hemarthrosis	40	0.03 (0.02-0.04)	56	0.05 (0.03- 0.06)
Urinary tract	59	0.05 (0.04-0.06)	80	0.06 (0.05 - 0.08)
All-cause mortality	104	0.08 (0.07-0.10)	239	0.19 (0.17 - 0.22)
Total knee arthroplasty (N = 132,726)				
Venous thromboembolism	261	0.2 (0.17-0.22)	456	0.34 (0.31 - 0.38)
Pulmonary embolism	222	0.17 (0.15-0.19)	403	0.30 (0.27 - 0.33)
Deep vein thrombosis	45	0.03 (0.02-0.04)	75	0.06 (0.04 - 0.07)
Arterial thromboembolism	323	0.24 (0.22-0.27)	556	0.42 (0.38 - 0.45)
Myocardial infarction	150	0.11 (0.09-0.13)	265	0.20 (0.18 - 0.22)
Ischemic stroke	108	0.08 (0.07-0.10)	194	0.15 (0.13 - 0.17)
Transient ischemic attack	67	0.05 (0.04-0.06)	103	0.08 (0.07 - 0.10)
Bleeding	925	0.70 (0.65-0.74)	1057	0.80 (0.75 - 0.84)
Procedural site	809	0.61 (0.57-0.65)	867	0.65 (0.61 - 0.70)
Gastrointestinal	32	0.02 (0.02-0.03)	41	0.03 (0.02 - 0.04)
Respiratory tract	10	0.01 (0-0.01)	25	0.02 (0.01 - 0.03)
Intracranial	a	a	a	a
Hemarthrosis	a	a	a	a
Urinary tract	66	0.05 (0.04-0.06)	91	0.07 (0.05 - 0.08)
All-cause mortality	156	0.12 (0.10-0.14)	362	0.27 (0.24 - 0.30)

a Numbers not shown due to privacy regulations by Statistics Netherlands (manages the Dutch Hospital Data registry) which prohibit reporting of numbers smaller than 10.

Venous thromboembolism risks following a first primary TKA procedure were 0.20% (0.17-0.22) at 30 days and 0.34% (0.31-0.38) at 90 days (Table 2). For ATE, these risks were 0.24% (0.22-0.27) and 0.42% (0.38-0.45). The bleeding risk at 30 days was 0.70% (0.65-0.74) and 0.80% (0.75-0.84) at 90 days. Lastly, all-cause mortality occurred in 0.12% (0.10-0.14) of patients at 30 days, and 0.27% (0.24-0.30) at 90 days.

### Temporal trends analysis

**Figure 2:**
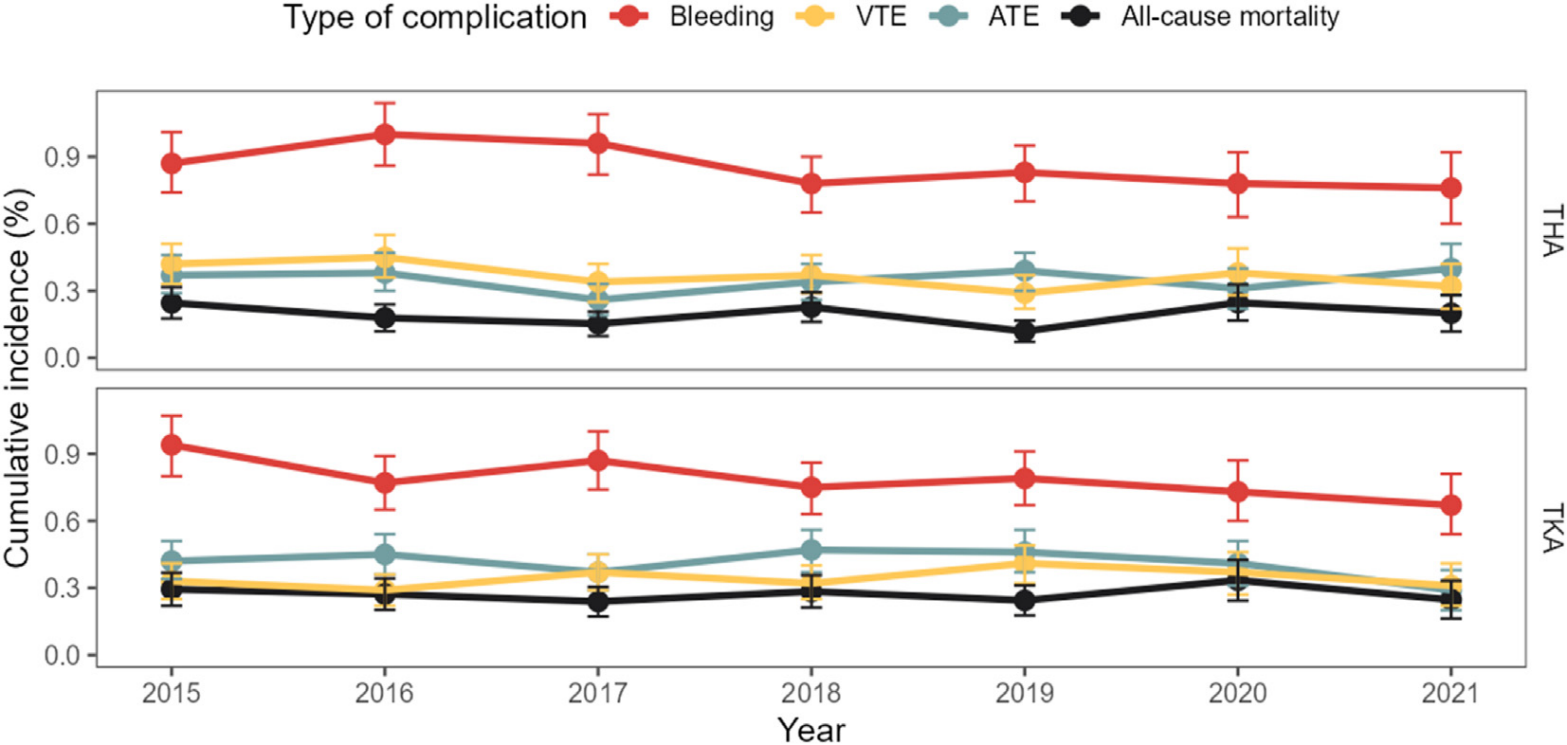
Time trend analyses: cumulative incidences at 90 days following arthroplasty surgery. THA, total hip arthroplasty; TKA, total knee arthroplasty.

For both THA, as well as TKA patients, risks remained approximately the same, even during the COVID-19 pandemic in 2020-2021 (Fig. 2). In addition, the distribution of outcomes was similar across all years and procedure types.

### Age- and sex-stratified risks

**Figure 3:**
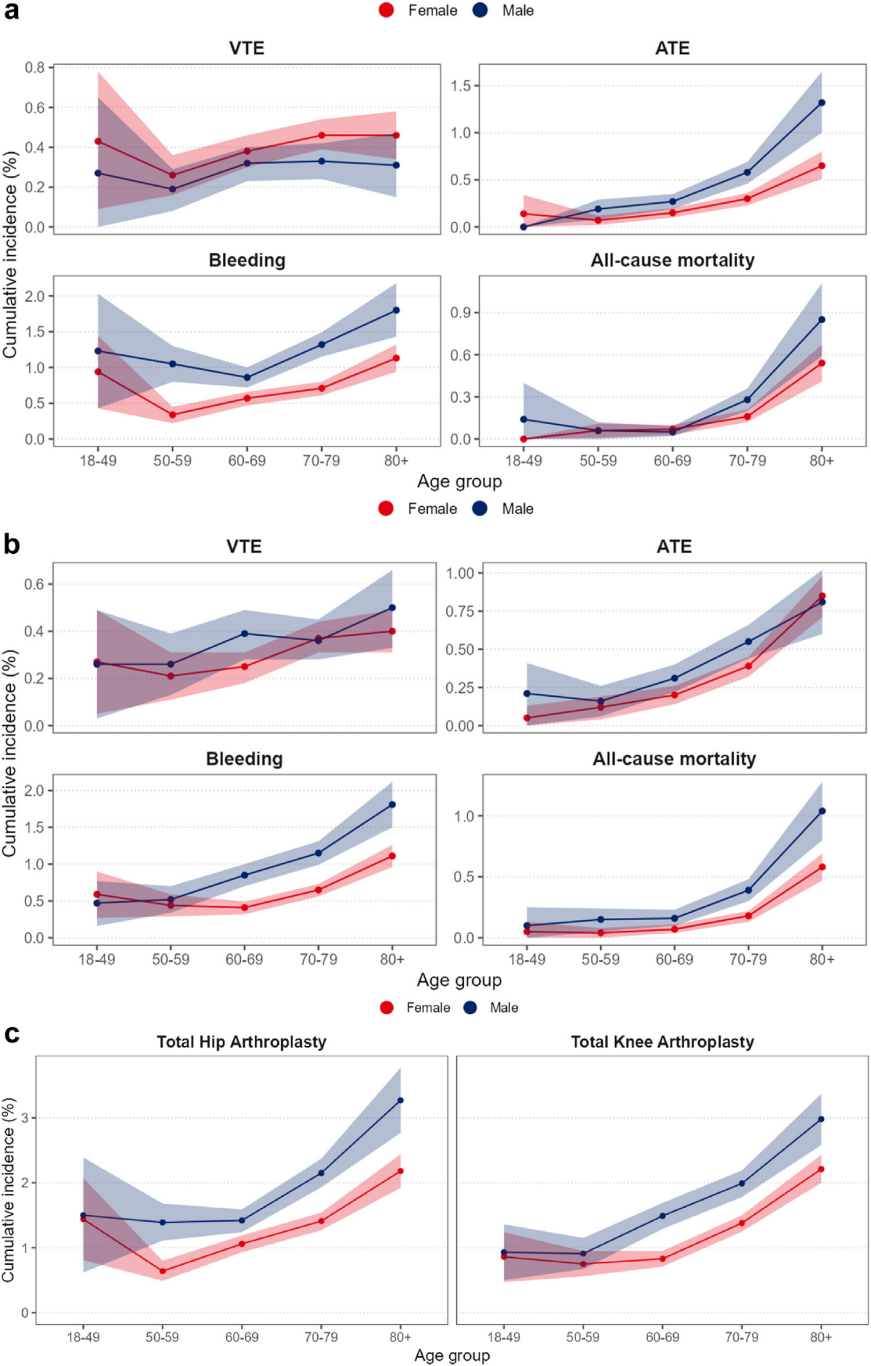
Age- and sex-stratified cumulative incidences at 90 days following arthroplasty surgery. (a) Total hip arthroplasty, (b) total knee arthroplasty, (c) combined cumulative incidence of major cardiovascular complications and all-cause mortality. VTE, venous thromboembolism; ATE, arterial thromboembolism.

The risk of VTE in women, following a THA procedure, ranged from 0.26% (0.16-0.36) to 0.46% (0.34-0.58) (Fig. 3a). In men, the risk of VTE was between 0.19% (0-0.65) and 0.33% (0.17-0.47). After TKA, the observed risk in women was between 0.21% (0.11-0.31) and 0.40% (0.31-0.49), while for men, this was between 0.26% (0.03-0.49) and 0.50% (0.33-0.66) (Fig. 3b). The 90-day VTE risk ratio between men and women was 0.74 (0.60-0.90) and 1.14 (0.94-1.37) for THA and TKA, respectively (Table 3).

**Table 3:** Relative risk of complications for men compared to women following a THA or TKA.

Complications	THA		TKA	
	Risk ratio (95% confidence interval)	*P*-value	Risk ratio (95% confidence interval)	*P*-value
VTE				
Women	1	-	1	-
Men	0.74 (0.60-0.90)	.003	1.14 (0.94-1.37)	.178
ATE				
Women	1	-	1	-
Men	1.76 (1.46-2.12)	<.001	1.12 (0.94-1.33)	.201
Bleeding				
Women	1	-	1	-
Men	1.70 (1.51-1.91)	<.001	1.59 (1.41-1.80)	<.001
All-cause mortality				
Women	1	-	1	-
Men	1.27 (0.99-1.64)	.064	1.35 (1.24-1.47)	<.001
Any complication				
Women	1	-	1	-
Men	1.43 (1.31-1.56)	<.001	1.71 (1.39-2.10)	<.001

VTE, venous thromboembolism; ATE, arterial thromboembolism.

After a THA procedure, the risk of ATE ranged from 0.07% (0.20-0.12) to 0.65% (0.51-0.80) in women, and from 0% (0-0) to 1.32% (1.00-1.65) in men. For TKA, the range in women was 0.05% (0-0.13) to 0.85% (0.71-0.98), and in men, it was 0.16% (0.06-0.26) to 0.81% (0.60-1.02). At 90 days, the ATE risk ratio for man vs women was 1.76 (1.46-2.12) for THA and 1.12 (0.94-1.33) for TKA.

For bleeding, the risk following a THA in women was between 0.34% (0.22-0.45) and 1.13% (0.94-1.32), while for men, this was between 0.86% (0.72-1) and 1.80% (1.43-2.18). Following a TKA, the risk was between 0.41% (0.32-0.49) and 1.11% (0.96-1.26) in women, and between 0.47% (0.16-0.77) and 1.81% (1.50-2.12) in men. Men had a 1.70 (1.51-1.91) higher risk of bleeding than women following a THA, and 1.59 (1.41-1.80) following a TKA, at 90 days postoperatively.

The risk of all-cause mortality for women following a THA ranged from 0% (0-0) to 0.54% (0.41-0.67). In men, this was from 0.05% (0.02-0.09) to 0.85% (0.59-1.11). Following a TKA, it was between 0.04% (0-0.08) and 0.58% (0.47-0.69) for women, and between 0.10% (0-0.25) and 1.04% (0.80-1.28) for men. The risk ratio for THA was 1.27 (0.99-1.64) at 90 days and 1.35 (1.24-1.47) for TKA.

Lastly, the combined complication risk in women undergoing a THA was between 0.64% (0.49-0.80) and 2.18% (1.92-2.44), and between 1.39% (1.11-1.68) and 3.27% (2.77-3.77) for men. After a TKA procedure, the risk range for women was 0.75% (0.56-0.95) to 2.21% (2.00-2.43), and it was 0.91% (0.67-1.15) to 2.98% (2.58-3.37) for men (Fig. 3c). The overall THA and TKA risk ratios for men compared to women were 1.43 (1.31-1.56) and 1.71 (1.39-2.10), at 90 days postoperatively.

## Discussion

We have reported the 30- and 90-day cumulative incidences for VTE, ATE, bleeding, and all-cause mortality in patients undergoing a first primary THA or TKA for osteoarthritis in the Dutch hospital population. It was found that these risks have been stable over recent years, and we estimated age- and sex-stratified risks. In these stratified analyses, we found that the overall risks of cardiovascular complications and all-cause mortality can be as high as 3% in the first 90 days following arthroplasty surgery for men aged 80+. Overall, at 90 days, men had a 1.43-times higher risk of any complication following a THA, and 1.71 following a TKA, compared to women.

The overall risk estimates for VTE were lower than expected based on existing literature [1,3,26]. It is unlikely that this difference can be (completely) explained by surgical and perioperative improvements. Rather it seems that, because the DHD does not register diagnoses made by general practitioners and at outpatients’ clinics, we missed these diagnoses in our data, and hence underestimated the risk of DVT. This assumption is strengthened by previous research that found the ratio of DVT to PE following THA/TKA is between 1.5 and 4, while in our study, we found twice as many PE events compared to DVT [3,26,27]. If we would extrapolate this ratio to our result, we should have found an incidence of VTE between 0.78%-1.55% and 0.76%-1.52%, at 90 days following THA and TKA, respectively. These extrapolated VTE risks are in line with previous population studies following THA/TKA [1,26,28]. This also means that the ∼3% overall cumulative incidence of any major cardiovascular complication and all-cause mortality for men in the highest age category is underestimated.

Our overall risk estimates for both ATE and all-cause mortality were either similar or lower than those in existing studies which estimated these risks for THA/TKA patients during early years [1,[28], [29], [30]]. For bleeding, because of the specific definition handled in this study, it is difficult to compare with other studies as most studies use their own definition of major bleeding or clinically relevant non-major bleeding. A recent study on Danish TKA patients, for example, defined major bleeding as either an intracranial or gastrointestinal bleeding and found the 90-day risk to be between 0.2% and 0.5%, dependent on the type of thromboprophylaxis [28]. Our estimates of bleeding are higher mainly due to the inclusion of surgical-site bleedings. However, the latter are only registered if these bleedings lead to an emergency room visit exceeding 4 hours or hospital admittance, so minor bleedings dealt with by the GP are not included.

Although we observed slight differences in the risk for each of the studied outcomes during the study years, there was no clear temporal trend. Next to this, we also did not see a change in the observed risk during the COVID-19 pandemic. This aligns with a previous study which found that, although there were less THA and TKA procedures performed for osteoarthritis, the distribution of comorbidity status of patients was the same as that before the pandemic [31].

The stratified risk estimates predominantly showed that older patients have a relatively high risk of major cardiovascular complications and all-cause mortality in the first 90 days postoperatively. An interesting finding was that both women and men in the 18-49 years age category, following a THA, have a non-negligible risk of VTE (especially considering the underestimation explained before). While there is some uncertainty about the estimated risks, for women between 18 and 49 years of age, this finding corresponds to the comparatively high risk of VTE for this age group in the general population, which has been attributed to temporal risk factors (ie, hormonal contraceptives and pregnancy) [32]. Another interesting finding is the relatively high risk of bleeding in the 18-49 years age group, in both women and men, following a THA. Because there are no previous studies on the absolute age- and sex-stratified risks of bleeding following a THA, we cannot provide further context to these estimates. Again, while there is some uncertainty around the estimates, it is good for orthopedic surgeons to be aware of these absolute risks. Lastly, we found that mainly patients (both women and men) above the age of 70 years are at risk of ATE and all-cause mortality, while below this age, risks are low. These findings concur with the conclusions from previous studies [4,33,34].

Our study has several strengths and limitations. The main strength of our study is that we could include a large, unselected nationwide population which allowed for relatively precise stratified risk estimates. Another strength is that we aimed to only include patients who had their first primary THA or TKA. This is important as we thought that patients who had a second THA or TKA would have a different risk compared to those undergoing a first: patients who undergo a second procedure must first have survived the first procedure, and second, a severely complicated (eg, by a myocardial infarction or a PE) first procedure will likely make the patient and treating physician hesitant to perform another arthroplasty. Hence, patients who undergo a second procedure will be a selective “healthier” subset of the entire THA and TKA population [24].

### Limitations

Identification was done on codes, and this allows for misclassification or, in the case of the procedures, missing an inclusion. To check whether our included number of procedures was accurate, we correlated this with the numbers reported by the Dutch Arthroplasty Register. Taking into account the procedures performed in private clinic, we included approximately 94% of registered THA procedures and 96% of registered TKA procedures [23].

Misclassification definitively occurred for the DVT diagnoses. Because DVT diagnoses, in the Netherlands, are often made by the general practitioner, and these are not included in the Dutch hospital registry, we misclassified patients as having no DVT while they actually had one. This led to an underestimation of DVT incidence, which resulted in an underestimation of the risk of VTE. Because of this, we presented the expected risks of VTE based on previous literature in combination with the incidence of PE in our cohorts. Although we expect that misclassification is less likely for the other complications, as these warrant hospital admissions or emergency room visits longer than 4 hours, we cannot be certain of this and hence reported cumulative incidence estimates could also be a (slight) underestimation of the actual risks.

Arthroplasty procedures performed in private clinics are not registered in the DHD. Because patients who undergo an operation at a private clinic generally have less comorbidity than patients operated in the hospital, our results will not be generalizable to procedures performed in private clinics.

## Conclusions

In conclusion, we found that the risks of VTE, ATE, bleeding, and all-cause mortality following a first primary THA or TKA for osteoarthritis remained stable over recent years. Furthermore, by estimating up-to-date age- and sex-stratified absolute risks for THA and TKA patients, we showed that the risk of cardiovascular complications and all-cause mortality is generally low. Orthopedic surgeons should be aware of the complication risk for men in the highest age category, which is at least 3%. With these results, orthopedic surgeons are better equipped to inform individual patients on their expected complication risks and target those patients in whom preventive measures outweigh potential side effects.

## Acknowledgments

The authors thank Qingui Chen for reviewing the syntax.

## Conflicts of interest

The authors declare there are no conflicts of interest.

For full disclosure statements, refer to https://doi.org/10.1016/j.artd.2024.101597.

## CRediT authorship contribution statement

**Mark J.R. Smeets:** Writing – original draft, Visualization, Project administration, Methodology, Formal analysis, Data curation, Conceptualization. **Maaike G.J. Gademan:** Writing – review & editing, Methodology, Conceptualization. **Rob G.H.H. Nelissen:** Writing – review & editing, Conceptualization. **Suzanne C. Cannegieter:** Writing – review & editing, Supervision, Methodology, Conceptualization. **Banne Nemeth:** Writing – review & editing, Supervision, Methodology, Conceptualization.

## Figures and Tables

**Appendix Table 1:** Procedural codes and ICD-10 codes used to identify total hip and total knee arthroplasties and the outcomes of interest.

Variables	Type of code	Code(s)
Pulmonary embolism	ICD-10	I26
Deep vein thrombosis	ICD-10	I80.1 - I80.3, I82.8, I82.9
Myocardial infarction	ICD-10	I21, I22
Ischemic stroke	ICD-10	I63
Transient ischemic attack	ICD-10	G45.9
Articular bleeding	ICD-10	M25.0
Procedural related bleeding	ICD-10	T81.0
Gastrointestinal bleeding	ICD-10	I85.0, K25.2, K25.4, K25.6, K26.0, K26.2, K26.4, K26.6, K27.0, K27.2, K27.4, K27.6, K28.0, K28.2, K28.4, K28.6, K29.0, K29.2
Respiratory tract bleeding	ICD-10	R04.0, R04.2, R048, R049
Intracranial bleeding	ICD-10	I60, I61, I62
Urinary tract bleeding	ICD-10	R31.9, N02
Hip osteoarthritis	ICD-10	M16
Knee osteoarthritis	ICD-10	M17
Total hip arthroplasty	ZA-codes	038567
	CBV-codes	038567, 338568, 338568A, 338568B, 338568J, 338568K, 338568L, 338568P, 338568R, 338568W
Total knee arthroplasty	ZA-codes	038663
	CBV-codes	038663, 338662C, 338662E, 338662T, 338662U, 338663C, 338663L, 338663R

ZA, “Zorgactiviteiten” (care activities); CBV, “Verrichtingenbestand”(care activities registry).

## Appendix A Supplementary data

Conflict of Interest Statement for Cannegieter

Conflict of Interest Statement for Gademan

Conflict of Interest Statement for Nelissen

Conflict of Interest Statement for Nemeth

Conflict of Interest Statement for Smeets
