# Supplementary material for: Age- and Sex-Specific Risks of Major Cardiovascular Complications and All-Cause Mortality Following Elective Hip and Knee Arthroplasty in the Netherlands: A Dutch Hospital Data Registry Study
Source: Arthroplast Today. 2025 Jan 3;31:101597. doi: 10.1016/j.artd.2024.101597 (PMC11754685; doi:10.1016/j.artd.2024.101597)
Supplement: Conflict of Interest Statement for Smeets [file mmc5.pdf]

INDIVIDUAL CONFLICT OF INTEREST STATEMENT

American Association of Hip and Knee Surgeons

(Adopted from the American Academy of Orthopaedic Surgeons disclosure statement)

The following form **must be filled out completely and submitted by each author (example, 6 authors, 6 forms). All items require a response. If there is no relevant disclosure for a given item, enter "*None.*"**

Age- and sex-specific risks of major cardiovascular complications and all-cause mortality following elective hip and knee arthroplasty in the Netherlands: a Dutch Hospital Data Registry study

1. Royalties from a company or supplier (The following conflicts were disclosed)

None

2. Speakers bureau/paid presentations for a company or supplier (The following conflicts were disclosed)

None

3A. Paid employee for a company or supplier (The following conflicts were disclosed)

None

3B. Paid consultant for a company or supplier (The following conflicts were disclosed)

None

3C. Unpaid consultants for a company or supplier (The following conflicts were disclosed)

None

4. Stock or stock options in a company or supplier (The following conflicts were disclosed)

None

5. Research support from a company or supplier as a Principal Investigator (The following conflicts were disclosed)

None

6. Other financial or material support from a company or supplier (The following conflicts were disclosed)

None

7. Royalties, financial or material support from publishers (The following conflicts were disclosed)

None

8. Medical/Orthopaedic publications editorial/governing board (The following conflicts were disclosed)

None

9. Board member/committee appointments for a society (The following conflicts were disclosed)

None

Each author must sign AND print or type his/her name, date and submit a separate form

In addition, one BLINDED Conflict of Interest form (no author names used) should be submitted per manuscript with all author disclosures.

31-10-2023

Author Name S.C. Cannegieter | Author Signature | Date
